# Global observational survey verifying surgeon utilization of the Validated Intraoperative Bleeding (VIBe) scale for use in clinical practice

**DOI:** 10.1016/j.sipas.2022.100123

**Published:** 2022-08-28

**Authors:** Pierre R. Tibi, Abe DeAnda, Steve KW Leung, Abel PH Huang, Terri Siebert, Stephen M. Dierks, Daniel M. Sciubba

**Affiliations:** a Department of Cardiothoracic and Vascular Services, Yavapai Regional Medical Center, Prescott, AZ, USA; b Division of Cardiovascular and Thoracic Surgery, University of Texas Medical Branch, Galveston, TX, USA; c Department of Urology, Western General Hospital, Lothian University Hospitals NHS Division, Edinburgh, UK; d Division of Neurosurgery, National Taiwan University Hospital & College of Medicine, Taipei, TW, USA; e Medical Affairs Advanced Surgery, Worldwide Medical Affairs, Baxter Healthcare Corporation, One Baxter Parkway, Deerfield, IL 60015, USA; f Department of Neurosurgery, Northwell Health, New York, NY, USA

**Keywords:** VIBe, Bleeding, Hemostasis, Scale, Surgery, Validated

## Abstract

**Introduction:**

Based on US-FDA guidelines to create tools for development and evaluation of medical devices in non-clinical and clinical studies, the Validated Intraoperative Bleeding (VIBe) Scale was developed. The current investigation expands upon the earlier validation study with multi-specialty surgeons to further corroborate these findings and establish feasibility to implement VIBe SCALE into clinical practice and surveys degree of anticipated bleeding across common procedures in multiple surgical disciplines.

**Methods:**

Following online didactic VIBe SCALE training, participating surgeons graded bleeding in ten porcine model videos corresponding to the original validation study. Surgeon and practice information were collected along with percentage of hemostatic product utilization in surgical procedures, the top three surgical procedures performed in their specialty, together with self-reported percentage of bleeding per VIBe SCALE bleeding grade typically encountered in those procedures.

**Results:**

Using a web-based interface, survey data were collected from 585 participating surgeons; 339 (58%) participants were from academic or university setting, 170 (29%) had >20 years of surgical experience, 217 (37%) performed 11–20 surgeries per month. Overall, 125 (21%) surgeons reported conducting up to 10% minimally invasive surgeries in their practice. Total, 334 (57%) participants considered patient's coagulation status while selecting intraoperative adjunctive hemostatic product, if required; majority (*n* = 552 [94%]) considered bleeding intensity important when selecting a hemostatic product intraoperatively. Interobserver concordance for VIBe SCALE of 0.89 (perfect concordance = 1.0) as a measure of reproducibility, was achieved.

**Conclusions:**

This survey strengthens the findings of the earlier validation study and provides human factors verification of the VIBe SCALE as a relevant tool to evaluate and assess bleeding severity across multiple surgical specialties, while demonstrating the useability in contemporary clinical practice and may facilitate hemostatic product choice.

## Highlights

• Clinical relevance of VIBe SCALE in surgery to choose hemostatic product.
• VIBe SCALE would be a good surgical team communication tool.
• Relevance of the VIBe SCALE bleeding grades in clinical practice.
• VIBe SCALE can assess bleeding severity across multiple surgical specialties.
• Results provide validation and human factors verification of the VIBe SCALE.

## Introduction

Annually, 100 million adults worldwide undergo inpatient non-cardiac surgery [1]. According to the Lancet Commission on Global Surgery, it is estimated that at least 4.2 million people die within 30 days of surgery each year, accounting for 7.7% of all deaths globally surpassed only by ischemic heart disease and stroke [2]. A recent large, prospective, global, multicenter cohort study of 40,004 patients showed that nearly half of all the deaths in non-cardiac surgical procedures were associated with three complications: major bleeding, cardiovascular complications, and sepsis, with median time of major bleeding happening the day of surgery [1]. In addition to the risk of mortality, morbidity from bleeding can also occur. Stokes et al., estimated that in the United States alone 1 out of 3 patients undergoing a surgical procedure (inclusive of cardiac surgery) has a bleeding related complication or requires a blood transfusion [3].

The Food and Drug Administration requires a validated bleeding scale to standardize bleeding outcomes to be used in clinical studies [4,5]. In view of this, in 2017, a Validated Intraoperative Bleeding (VIBe) Scale was developed to represent the full spectrum of blood loss and validated by 102 surgeons from multiple surgical specialties across 3 surgical cavities: thoracic, abdominal, and pelvic [5].

Developed based on videos of known bleeding rates in a porcine model, anatomical appearance, intervention, qualitative description, and visual estimation of rate of blood loss, the VIBe SCALE consists of 5 Grades (Grades 0 to 4) (Supplementary Fig. 1), where Grade 0 refers to no bleeding (≤1 mL/min blood loss) and Grade 4 refers to life threatening bleeding (>50 mL/min blood loss). VIBe focuses on intensity of blood loss irrespective of source and coagulopathic conditions. It was also shown that surgeons repeatedly and reproducibly assessed bleeding severities across different surgical cavities, regardless of specialty [5]. VIBe characterizes a surgeon's awareness of bleeding severity among all bleeding scenarios faced clinically, regardless of amount, rate, source, location or accessibility.

The recently conducted survey was executed globally with surgeons across multiple surgical specialties (General surgery, Neurosurgery, Gynecology, Cardiac surgery, Urology and Spine [both Neurosurgical and Orthopedic]) with two primary objectives established. First, to provide verification regarding perception and relevance of the VIBe SCALE bleeding grades, and second does this correlate to clinical practice. The online survey included review of training videos from the original VIBe SCALE where 585 surgeon participants provided further confirmation of the original validated scale. Secondly, surgeons provided input on their perceived severity bleeding seen in routine procedures based on their clinical practice. Intervention strategies for treating surgical bleeding were not included in this survey. However, the anticipated bleeding severity according to VIBe may support proactive surgical planning in respect to bleeding events.

## Materials and methods

The purpose of this survey was to perform a human factors verification on the bleeding severity scale for use in clinical practice across a considerably larger international group of surgeons that included a broader group of specialties as well as regional practice variations not represented in the initial survey. This survey included board-certified surgeons with a minimum of 5 years postgraduate surgical experience. They were recruited from academic, public, private, or non-profit hospital settings.

Surgeons were identified based on demonstrated involvement or interest in clinical studies of hemostatic agents. Surgeons were not selected based on previous consultancy agreements, product usage, or industry affiliations. Participating surgeons were not compensated for this survey.

In doing so, surgeon responses were prospectively collected in a single session to assess the ability of surgeons to complete stream-lined web-based didactic training of the VIBe SCALE and video reviews of assessing the 5 bleeding grades. Following the online review and training of VIBe, participants were then asked to score ten porcine bleeding model videos from the original validation study [5]. All 585 participating surgeons (except Spine) graded the same 10 video based on VIBe examples of bleeding similar to types of bleeds they may see in their practice. This was performed as described in the original validation study by Lewis et al. [5]. Participants who identified as Spine surgeons scored a different set of 10 videos relevant to their practice. The scored videos represented the range of VIBe SCALE severities and included bleeding from bone, mucosal lesions, diffuse muscular and soft tissue sources, various organs with differing insults as well as open vascular hemorrhage.

Additionally, participants provided information regarding demographics, background, training, procedure and practice profile, hemostasis management based on patient coagulation status, percentage of patients treated with anti-platelet/ anticoagulants (pre- and post-operation), top three surgical procedures in their specialty that contributed to the majority of their practice where they used adjunctive hemostatic products and self-reported perceived VIBe grade of bleeding encountered in those common procedures (Table 1).

**Table 1:** Demographics and baseline characteristics of participating surgeons.

Parameters	n (%)
**Total Number of Participating Surgeons**	585
**Specialties**	
General Surgery	184 (31)
Neurosurgery	117 (20)
Gynecology	110 (19)
Cardiac Surgery	87 (15)
Ortho and Neuro Spine	57 (10)
Urology	30 (5)
**Region**	
Asia Pacific region	271 (46)
European region	243 (42)
American region	71 (12)
**Type of Hospital Practice**	
Academic/University	337 (58)
Academic/University/Private Community Hospital	7 (1)
Academic/University/Private Community Hospital- Public	18 (3)
Academic/University Public	40 (7)
Private/Community Hospital	73 (12)
Private/Community Hospital Public	8 (1)
Public	102 (17)
**Years in Surgical Practice**	
0–5 years	75 (13)
6–10 years	98 (17)
11–15 years	132 (23)
16–20 years	112 (19)
>20 years	168 (29)
**Total Surgical Procedures per Month**	
1–10	82 (14)
11–20	216 (37)
21–30	166 (28)
>30	121(21)
**Percentage of Total Surgical Procedures that are MIS**	
0	29 (5)
0.1–10	125 (21)
11–20	78 (13)
21–30	76 (13)
31–40	40 (7)
41–50	66 (11)
51–60	39 (7)
61–70	33 (6)
71–80	52 (9)
81–90	30 (5)
91–100	17 (3)
**Percentage of Surgical Procedures Where an Adjunctive Hemostatic Product May Be Used**	
1%−25%	255 (44)
26%−50%	153 (26)
51%−75%	44 (8)
76%−100%	133 (23)
**Percentage of Patients Treated with Anticoagulant/Antiplatelet Therapy During Preoperative Phase**	
0%−20%	218 (37)
20%−40%	160 (27)
40%−60%	99 (17)
60%−80%	52 (9)
80%−100%	56 (10)
**Percentage of Patients Continuing Anticoagulant/Antiplatelet Therapy Postoperative Phase**	
0%−20%	180 (31)
20%−40%	127 (22)
40%−60%	88 (15)
60%−80%	63 (11)
80%−100%	127 (22)
**Does Patient Coagulation Status Determine the Type of Intraoperative Adjunctive Hemostatic Product Used**	
Yes	334 (57)
No	251 (43)
**Does Bleeding Intensity Determine the Type of Intraoperative Adjunctive Hemostatic Product Used**	
Yes	552 (94)
No	33 (6)

All percentages are rounded off to nearest whole number for ease in calculation.

Based on the previous study validated by 102 surgeons from multiple surgical specialties across 3 surgical cavities: thoracic, abdominal, and pelvic [5], the current survey was designed to include at least 30 surgeons per surgical specialty, targeting a minimum of 150 surgeons across at least six different surgical specialties, which included General surgery, Cardiac surgery, Neurosurgery, Gynecology, Urology and Spine [both Neurosurgical and Orthopedic].

All survey data were entered into a web-based interface allowing “yes-no,” “multiple choice” and “free text” responses. Descriptive statistics for the participants’ responses were tabulated according to surgical specialty, frequency of procedures, and self-reported demographics. Qualitative data were summarized using frequencies and percentages.

## Results

### Demographics and baseline characteristics

This global survey was conducted from 01-Sep-2020 to 07-Jun-2021. Responses from 585 participating surgeons (Asia Pacific region: *n* = 271 [46%], European region: *n* = 243 [42%], North and South America: *n* = 71 [12%]) were collected using a web-based interface or in-person interviews. Majority of the surgeons were from academic or university setting (*n* = 337 [58%]). There were a greater number of General surgeons (*n* = 184 [31%]) followed by Neurosurgeons (*n* = 117 [20%]), Gynecologists (*n* = 110 [19%]), and approximately 15% (*n* = 87) were Cardiac surgeons (Table 1). A total of 280 (48%) out of 585 surgeons had more than 15 years of surgical experience and 49% (*n* = 287) performed more than 20 surgeries per month. Only 39% (*n* = 232) of the total surgeons reported that no more than 20% of their practice consisted of minimally invasive surgeries (Table 1).

### Concordance of VIBe bleeding SCALE

After training, the majority of the surgeons 357 (61%) scored between 70 and 90%, and 94 (16%) of the surgeons scored a full 100% on the test videos (Table 2). The agreement among surgeons (interobserver agreement) measures reproducibility across multi-specialties. Interobserver agreement was analyzed using the Kendall coefficient of concordance (Kendall's W) statistic. A Kendall's W of ≥ 0.7 is considered “acceptable”, ≥ 0.8 as “appreciable”, ≥ 0.9 as “excellent”, and 1.0 as “perfect” concordance. The overall interobserver agreement among all surgeons in this larger sample was 0.89 for all types of videos, constituting “appreciable” concordance further supporting VIBe's usability, clarity and relevance to evaluate bleeding severity clinically and consistently across surgical specialties in open and minimally invasive surgery (MIS).

**Table 2:** Overall VIBe video scores of participating surgeons (*n* = 585).

Overall Score	Number of Surgeons: n (%)
Score 50% or less	52 (9%)
Score 60%	82 (14%)
Score 70–90%	357 (61%)
Score 100%	94 (16%)

### Utilizing the VIBe scale in clinical practice

In this survey, 63% (*n* = 369) of the surgeons reported that their patients received pre-operative anticoagulant/antiplatelet therapy in some or all of the procedures, while 48% (*n* = 281) continued anticoagulant/antiplatelet therapy post-operatively in 40% to 100% of their patients. Many participating surgeons (*n* = 408 [70%] confirmed the utilization of adjuvant hemostatic agent in up to 50% of their surgical procedures (Table 1).

**Fig. 1:**
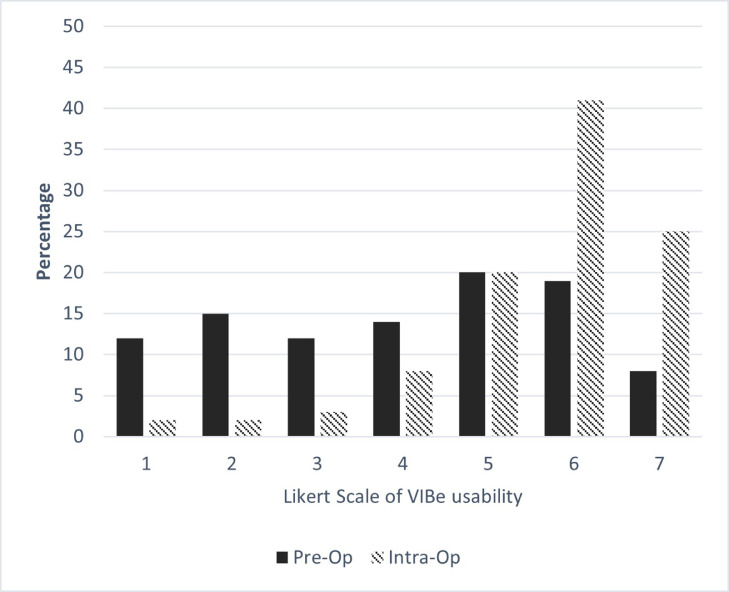

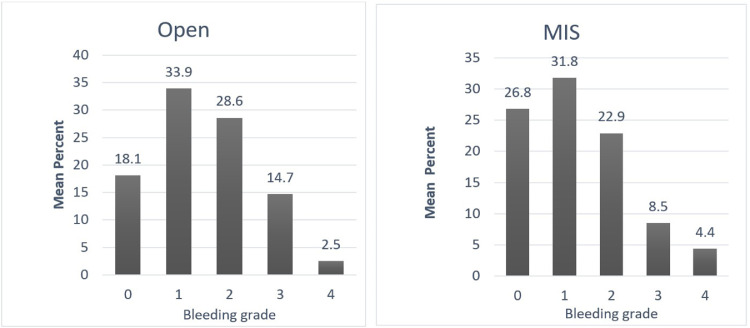
A: Grading* of Utility of VIBe Scale as a Tool in Preoperative and Intraoperative Stages *On a scale of 1–7 with 1 being low and 7 being high, participants rated their ability to utilize VIBe as a tool preoperatively and intraoperatively. B: Mean Percentage Value of Perceived Bleeding Grades by Participating Surgeons (*n* = 585) in Total Overall Surgical Procedures for Open vs. MIS.

When survey contributors were asked to rate their ability to utilize the VIBe SCALE as a tool in preoperative stage on a scale of 1 to 7 with 1 being lowest and 7 being highest, 61% of the participating surgeons (*n* = 354) rated VIBe as 4 or greater and suggested that knowing the bleeding potential of the patient would help them to create a plan, communicate potential blood loss and be prepared for bleeding during surgery. Surgeons were then asked to rate the applicability of VIBe as an intraoperative tool and a majority (*n* = 547 [94%]) rated VIBe as 4 or greater, (86% [*n* = 501] rated it as 5 or greater) and considered VIBe as a reliable and efficient tool to assess and communicate bleeding during the different stages of the procedure (Fig. 1A). Several surgeons commented that the bleeding scale would be a good surgical team communication tool.

### Real-world perception by surgeons regarding bleeding severity across

#### Multi-specialties

Bleeding intensity was surveyed across multiple specialties. Common cases and expected VIBe bleeding severity were also captured. Overall, 585 surgeons from 6 specialties provided bleeding intensity responses for minimally invasive as well as open surgery. From the survey, the analysis of their reported bleeding perception noted in their practice showed that regardless of approach or type of surgery, significant bleeding was identified across the spectrum of surgical subspecialties in both open and minimally invasive procedures. Their real-world perception of bleeding in over 50% of procedures in either open or minimally invasive approach were perceived to be Grades 1 and 2. When Grade 3 was added to the bleeding analysis, significant bleeding was observed in over 75% of open procedures and over 60% in minimally invasive procedures (Fig. 1B). Due to self-reporting of procedures based by specialty, the cases for cardiac surgery were all open surgeries, and MIS was not represented in this sub-specialty.

**Fig. 2:**
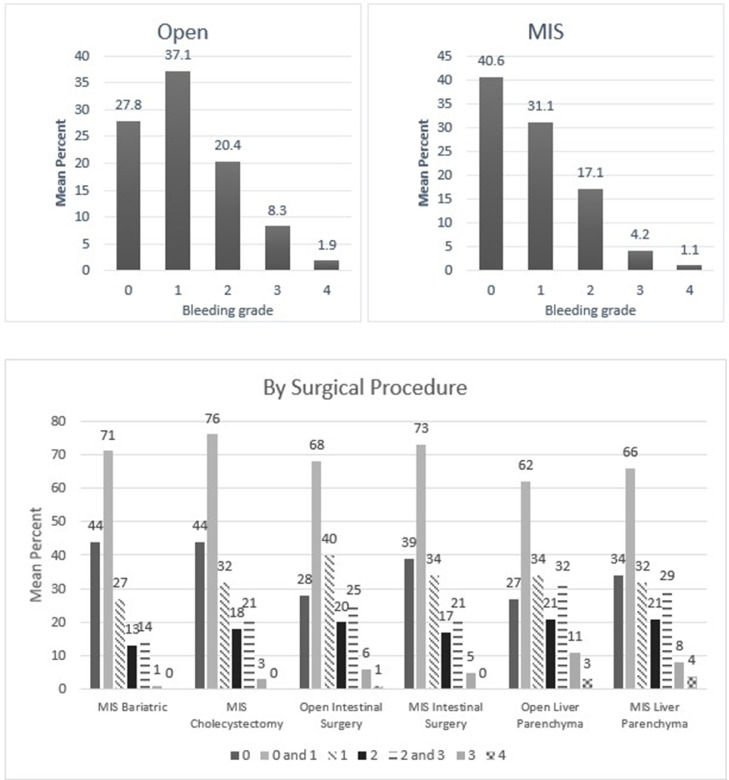
Mean percentage value of perceived bleeding grades by participating surgeons (*n* = 184) in surgical procedures of general surgery.

**Fig. 3:**
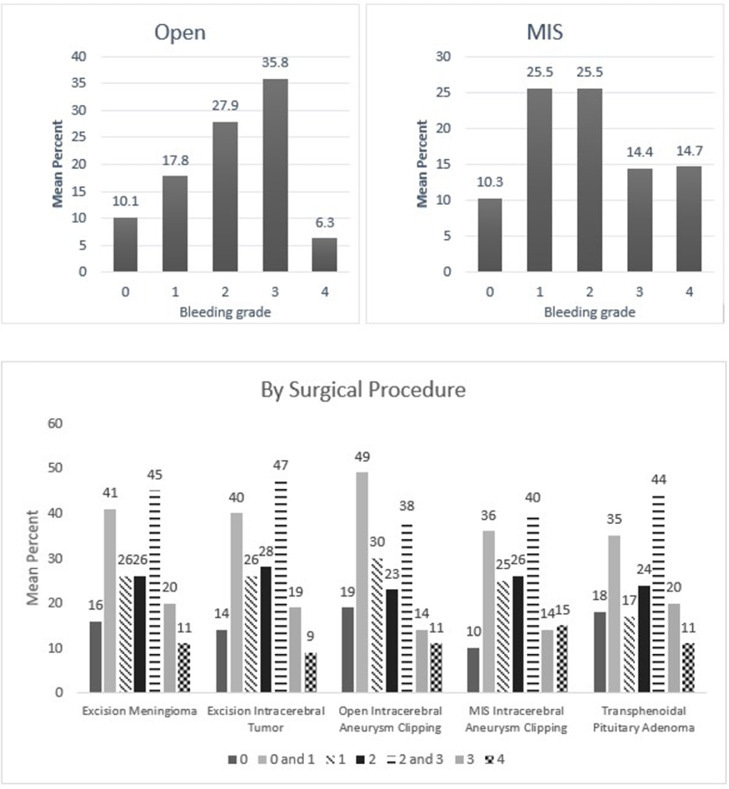
Mean percentage value of perceived bleeding grades by participating surgeons (*n* = 117) in surgical procedures of neurosurgery.

**Fig. 4:**
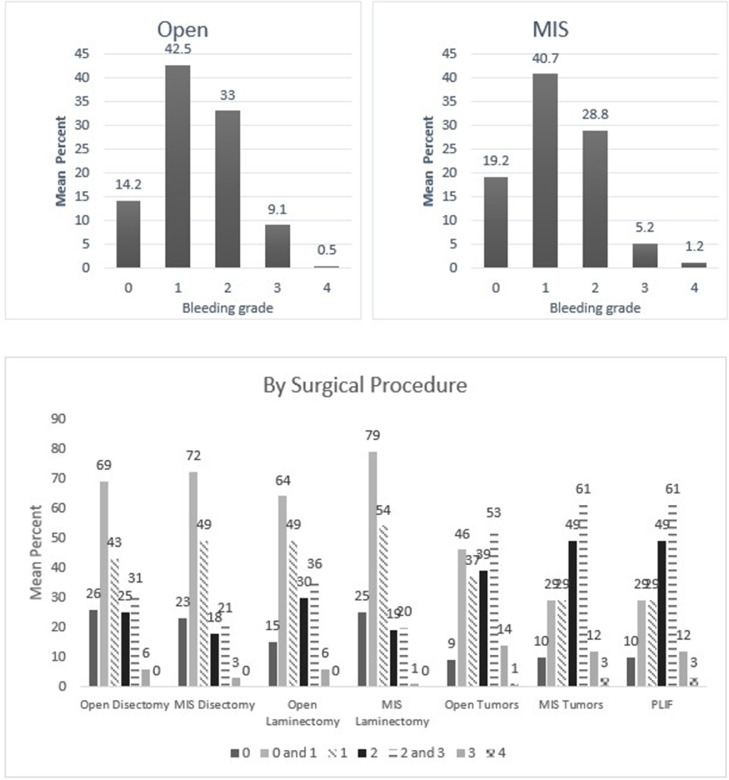
Mean percentage value of perceived bleeding grades by participating surgeons (*n* = 57) in surgical procedures of orthopedic and neurological spine.

**Fig. 5:**
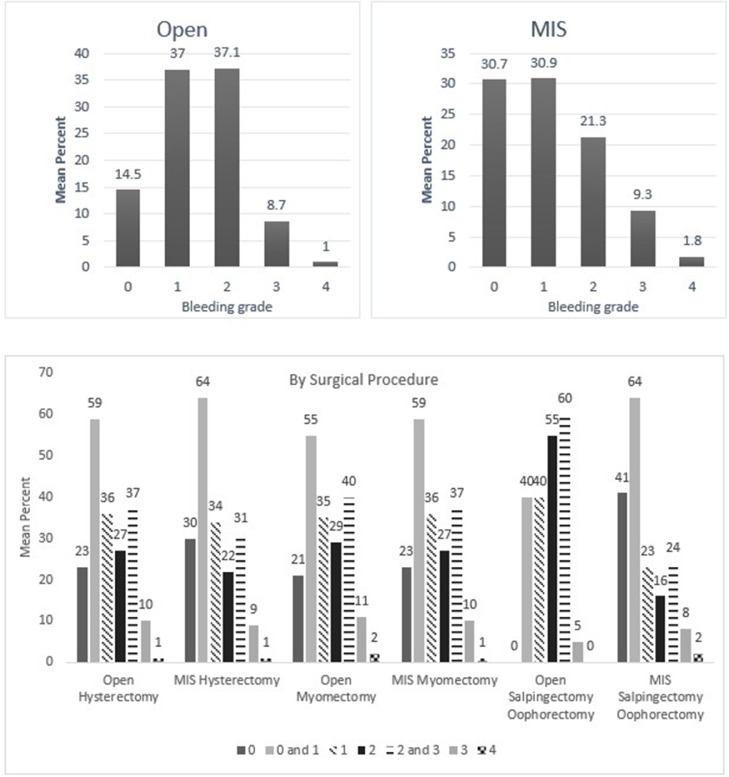
Mean percentage value of perceived bleeding grades by participating surgeons (*n* = 110) in surgical procedures of gynecology.

**Fig. 6:**
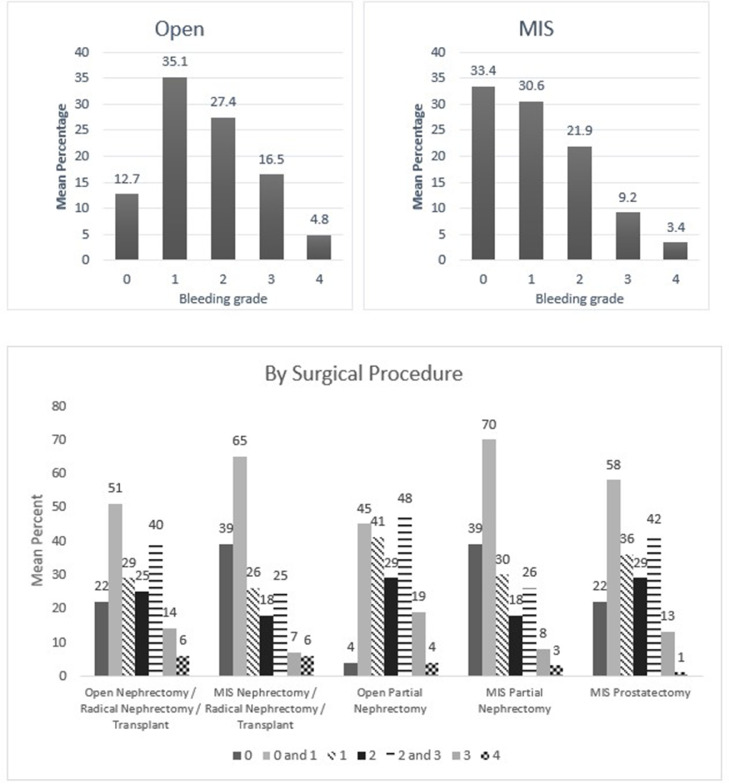
Mean percentage value of perceived bleeding grades by participating surgeons (*n* = 30) in surgical procedures of urology.

**Fig. 7:**
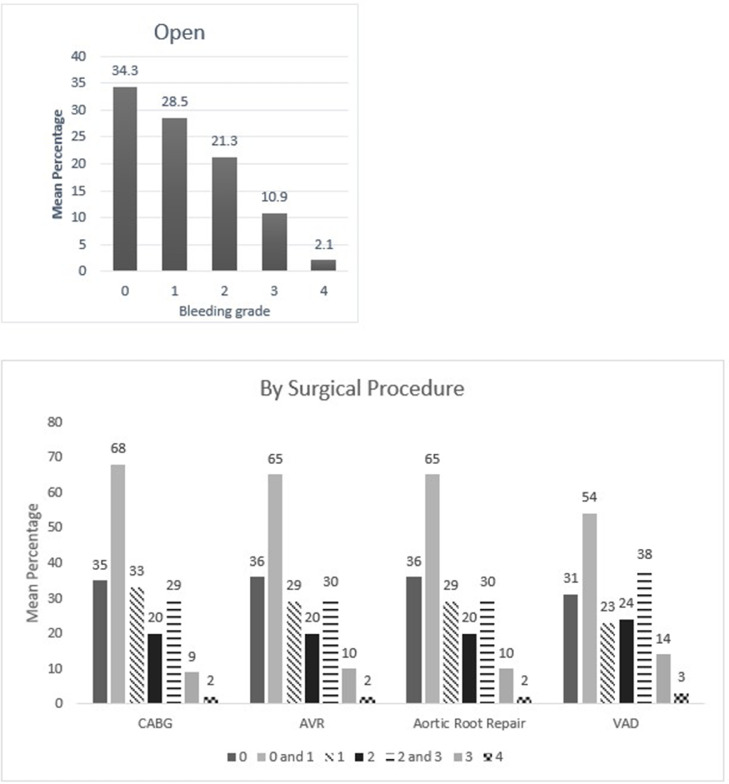
Mean percentage value of perceived bleeding grades by participating surgeons (*n* = 87) in surgical procedures of cardiac surgeries.

When these self-reported responses were analyzed, surgeons had described encountering variable intraoperative bleeding across surgical subspecialties and throughout the duration of the case. The breadth of bleeding was most prominent in cranial and spinal tumor resections, aneurysm interventions (Neurosurgery & Spine), laminectomies (Spine), hepatic surgeries (General Surgery), aortic and ventricular access device interventions (Cardiac Surgery), uterine surgeries (Gynecology), partial nephrectomies and prostatectomies (Urology). The outcomes demonstrated significant yet variable bleeding intensity throughout the cases, highlighting the need for different hemostasis options being available for clinicians during these routine operations. These results are shown in Fig. 2, Fig. 3, Fig. 4, Fig. 5, Fig. 6, Fig. 7.

## Discussion

Currently, there exists no validated and widely accepted scoring system which can quantify bleeding severity during surgical procedures. The applicability of the 5-point Likert type scale developed by Siegel et al. to evaluate the intra-operative bleeding in stapling applications is limited to harmonic scalpels [6]. Another 6-point pictorial bleeding scale developed by Adams et al. has limited usability beyond the lesion type described in preclinical studies [7], [8], [9]. In the recently updated Society of Thoracic Surgeons (STS) Practice Guidelines, Tibi et al., stated that “*the development of intraoperative bleeding scales (Lewis 2017) may be helpful in determining which hemostatic agent is more likely to be useful in certain situations, but nevertheless, the source of bleeding and the patient's coagulation profile are important factors that may preclude the actions of any and all topical hemostatic agents. Assessment of topical hemostatic agents in clinical RCTs is extremely difficult due to difficulty in establishing reliable end points and using a reproducible bleeding scale intraoperatively may be the best method to compare efficacy of topical hemostatic agents*” [10]. In another recent published large retrospective study, Iannitti et al., discussed that adjunctive topical hemostatic products are used in addition to primary methods (mechanical and thermal) to control bleeding [11]. Hemostatic agents are generally classified as active or passive depending on how they interact with the coagulation cascade. The outcomes of the Iannitti study showed the use of active hemostatic products alone was associated with significant lower rates of bleeding-related complications, shorter intensive care unit length of stay, reduced overall length of stay and lower total hospital costs compared with combined use of passive and active hemostatic products. The profile of patients undergoing surgery has changed over the years with increasing age and comorbidities [12,13]. With the increase in use of antiplatelet/anticoagulation products in this cohort, the interaction between hemostatic products in the presence of anticoagulation agents has been investigated. One preclinical study using a heparinized porcine model, investigated combining the visual presentation of VIBe and selection of a passive or active hemostatic product to efficiently select the appropriate hemostatic agent for fast and consistent hemostasis [14]. A treatment approach which considers critical bleeding-related factors such as severity, risk and variability based on surgery type combined with the use of a standardized bleeding severity assessment tool may provide guidance to surgeons in choosing the optimal hemostatic product to improve surgical outcomes and cost [11].

The VIBe SCALE is the first validated intraoperative bleeding severity scale that fulfills the US FDA criteria, developed to provide the ability for standardization between topical hemostatic agent clinical studies, labeling claims and potential to mitigate risk to patients by defining use of products as out of scope for clinical situation based on their efficacy [5].

The current observational survey is the largest global survey done to date which studies the perception of bleeding severity in real world scenario using VIBe. This survey was undertaken to further strengthen the findings of the earlier validation study conducted in 127 surgeons of three different specialties, including spinal surgery [5,15], and to explore the value of implementation of VIBe in clinical practice across multiple surgical specialties. This survey was conducted in a diverse and international population of surgeons across multiple specialties from both academic, as well as public and private hospital settings. Over 168 participating surgeons had more than 20 years of surgical experience and majority of participating surgeons performed 11 to 30 surgeries per month. In this survey, the robust data demonstrates VIBe is applicable to real world clinical practice.

One of the points discussed in the earlier validation study was whether the VIBe SCALE could appropriately classify blood loss in minimal invasive surgeries [3]. In the current survey, participating surgeons could confidently perceive the blood loss in minimally invasive procedures regardless of specialties using the VIBe SCALE **(**Fig. 2, Fig. 3, Fig. 4, Fig. 5, Fig. 6, Fig. 7). Additionally, the interobserver concordance (Kendall's coefficient or Kendall's W) of 0.89 in this study, with nearly 6 times the number of surgeons representing an even more diverse group of specialties, is “appreciable” and comparable to that found in the initial Lewis et al. study (Kendall's *W* = 0.91) (5) and the subsequent Sciubba et al. spine group (Kendall's *W* = 0.88) [15]. In a separate Spanish multicenter study evaluation of the VIBe SCALE for use in hepatobiliary (HPB) surgery, Ramia et al. found that 47 HPB surgeons achieved an interobserver concordance of 0.929 [16]. This further strengthens the initial validation regarding agreement amongst surgeons across specialties: that severity of bleeding is uniformly recognized and categorized by VIBe.

VIBe quantifies estimated real-time blood loss irrespective of anatomical source and coagulation status [5]. This survey provides additional evidence that VIBe is simple and intuitive to learn via an online tool with minimal investment for institutions and can be implemented across diverse surgical procedures with consistent, reproducible results over the range of bleeding severity grades [15]. These qualities should encourage additional studies to establish VIBe as a Medical Device Development Tool appropriate tool for bleeding severity as recommended by FDA guidance [4].

Since this was a diverse group of participating surgeons from academic, public, private and/or non-profit hospital settings, data obtained from this survey is both robust and reliable. Participants self-reported the grade of bleeding they encounter in certain common surgical procedures. In addition, the expected bleeding severity was estimated over the course of each type of surgery, which may enhance the ability for clinical application of VIBe and efficiently select and differentiate the appropriate hemostatic product [15].

When surveying the different specialties and stratifying VIBe bleeding severity temporally for a group of diverse open and minimally invasive surgical cases, it is anticipated this will provide several potential new opportunities. Based on certain critical points of a procedure, the entire surgical team may be able to anticipate the degree of blood loss at important points in a procedure and use this as a good communication tool intra-op as well as a pre-operative planner. This may also provide post-operative management guidance and enhance better blood product management for the duration of the post-operative course.

This survey provides additional evidence that supports the initial studies, and that development of a universal bleeding scale across multiple surgical specialties is both needed and feasible to provide a necessary tool in the development of better patient care related to surgical bleeding for clinicians, researchers and regulatory authorities. Choice of hemostat based on degree of VIBe bleeding in each case was not investigated in this survey, however, use of a standardized bleeding severity assessment tool may provide guidance to surgeons in choosing the optimal hemostatic product to improve surgical outcomes [11,14]. The findings of this real-world observational survey may encourage trials across additional surgical specialties to further explore the useability of VIBe in clinical practice.

## Limitations

Since surgeons provided their own real-world experience, this could be considered a limiting factor in this observational survey. Although 585 surgeons were interviewed across the three globally diverse regions (Asia Pacific, Europe and the Americas) not all were equally represented, and participant numbers amongst the six surgical specialty groups were not proportionately balanced. Additionally, not all surgical specialties were represented, which could contribute to a selection bias. However, this larger and more diverse group of surgeons showed a similar interobserver concordance (0.89), consistent with prior studies with a more limited number and less diverse group of surgical specialists [5,15,16], which strengthen these current results. This further supports the usability, clarity and relevance of VIBe to evaluate bleeding severity consistently in both open and minimally invasive (MIS) procedures.

## Conclusion

This survey strengthens the findings of the earlier validation study and provides human factors verification of VIBe to assess bleeding severity across multiple additional surgical specialties, while demonstrating the useability and clinical relevance to facilitate and differentiate hemostatic product or method of choice. VIBe can be useful to help surgeons communicate anticipated hemostatic needs throughout the course of a case, enhancing efficiency which will provide a pathway to better outcomes. Utilization of VIBe may be an important avenue to not only critically evaluate hemostatic methods as recommended by the FDA but assist the team with planning hemostatic and blood product management.

## Funding support

This study was supported by Baxter Healthcare Corporation; the sponsor also funded the development of this manuscript. Drs. DeAnda, Huang, Sciubba, Leung and Tibi, did not receive compensation for drafting, reviewing, or otherwise participating in this publication. All authors made direct substantial contributions and provided critical review.

## Declaration of Competing Interest

Terri Siebert, RN and Dr. Stephen Dierks are both employees of Baxter Healthcare Corporation, Dr. Daniel Sciubba is a consultant with for Depuy-Synthes, Medtronic, Stryker, and Baxter Healthcare Corporation, Dr. Steve Leung has received honoraria from Baxter Healthcare Corporation and Olympus Medical, Drs. Abe DeAnda Jr, Abel PH Huang and Pierre Tibi have no conflicts of interests to declare.

## Appendix Supplementary materials

Video 1: VIBe SCALE Grade 0

Video 2: VIBe SCALE Grade 1

Video 3: VIBe SCALE Grade 2

Video 4: VIBe SCALE Grade 3

Video 5: VIBe SCALE Grade 4

Supplementary Fig. 1: VIBE Bleeding Scale Image, image 1
