# Supplementary figures and images for: Global observational survey verifying surgeon utilization of the Validated Intraoperative Bleeding (VIBe) scale for use in clinical practice
Source: Surg Pract Sci. 2022 Aug 28;12:100123. doi: 10.1016/j.sipas.2022.100123 (PMC11750003; doi:10.1016/j.sipas.2022.100123)

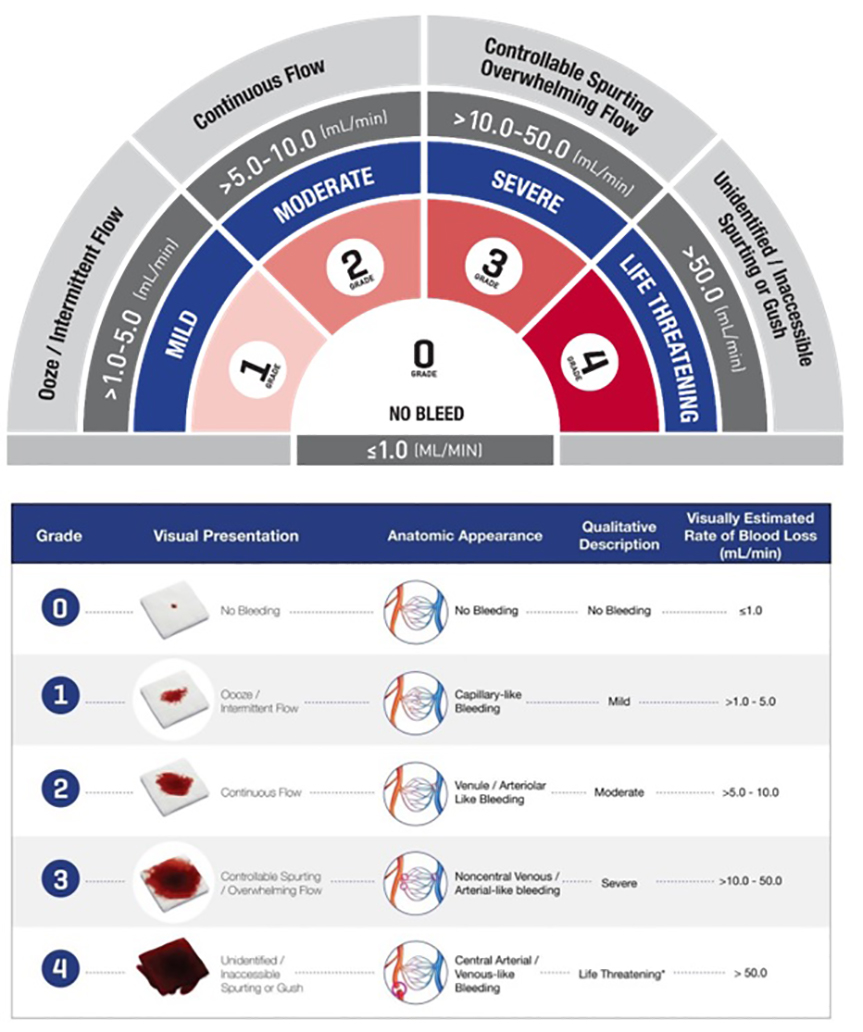

Supplement: Supplementary file 6 — Supplementary Fig. 1: VIBE Bleeding Scale [file mmc6.jpg]
